# Hospital Practice

**Published:** 1828-04-01

**Authors:** 


					1828] ( 529 )
rtetope;
OR,
CIRCUMSPECTIVE REVIEW.
1 .Ore trahit quodcunque potest, atque addit aceryo."
[8th MARCH, 1828.]
HOSPITAL PRACTICE!
1. HOPITAL COCHIN.
M. BoUI LLAUD ON DISEASES OF THE
Biliaky Ducts.
In a former number of this Journal, we
presented our readers with some inter-
esting facts relative to this point of pa-
thology, from the work of M. Andral,
the younger :?we shall now bring for-
ward some observations from a zealous
cultivator of pathological science, M.
Bouillaud.
Among the alterations of structure ob-
served in the biliary ducts, some are
common?others rather peculiar. In the
former class, we may reckon inflamma-
tion and its consequences, as induration,
suppuration, ulceration, thickening, &c.
Also, dilatation, contraction, and obstruc-
tion. Of those lesions which are some-
what peculiar to the parts in question,
are biliary concretions, and various other
morbid changes which the bile itself
undergoes, as well in its consistence as
in its chemical composition. It is but
seldom that we find any one of these
lesions uncomplicated with one or more
of the others. Thus, inflammation of the
internal membrane may produce contrac-
tion or even obliteration of the ducts?
and the presence of biliary calculi may
induce inflammation, and so forth.
The symptoms indicative of different
lesions in the excretory apparatus of the
liver are far from being satisfactorily
ascertained. Hence the greater neces-
sity for accumulating such facts as may
enable future observers to distinguish
diseases that are, in their nature, ob-
scure,.though, in their eflects, extremely
distressing and embarrassing.
Case 1. Peter Voisenat, aged 50 years,
rather embonpoint, entered the Cochin
Vol. VIII. No 16.
Hospital, on the 12th March, presenting
the following phenomena. He stated that
he had been ill about three months. The
skin and the conjunctiva were of an
orange colour?there was evident fluctu-
ation in the abdomen?but no infiltration
of the lower extremities. The tongue
was red?the pulse febrile. This fever-
ish state persisted for three weeks, at
which time, coma supervened, and the
patient died.
Dissection. On the inferior surface of
the liver, a tuberculous mass presented
itself, enveloping the situation of the gall-
bladder, not a vestige of whose parietes
could be found, by the most minute in-
vestigation. Neither could there be dis-
covered any trace of the ductus cysticus,
hepaticus, or choledochus. There was a
cavity or pouch in the situation of the
gall-bladder, containing a purulent fluid,
in which were several biliary concretions.
This pouch was adherent to the arch of
the colon, and here there was a com-
mencing ulceration which would soon
have opened a communication with the
intestine, and through which, no doubt,
the biliary calculi would have been dis-
charged. The vena portse was completely
obliterated.
Case 2. B. Lebant, 68 years of age,
having experienced severe and long-cor-r
tinued mental anxiety, entered the hos-
pital on the 4th November. On exami-
nation with the stethoscope, it was ascer-
tained that she had valvular disease of
the heart, especially of the left auriculo-
ventricular opening, of which she died a
few days after she was admitted. During
this period, there was not any symptom
evinced which led to the supposition of
disease about the excretory ducts of the
liver. On dissection, the convey surface
3 M
530 Periscope; or, Circumspective Review. [March S
of this organ was found adherent to the
abdominal parietes. The edge of the
liver descended as low as the iliac fossa
on the right side. The substance of the
organ was rather gorged with blood, but
otherwise healthy. The gall-bladder con-
tained 90 gall stones, of polished surface,
light yellow colour, and various magni-
tudes. The parietes of the biliary recep-
tacle were thickened?its internal mem-
brane red, and covered with mucus.?
There was bile in the small intestines.
In this case there was no pain com-
plained, of in the hepatic region?nor was
there any jaundice. The auriculo-ven-
tricular opening was contracted and puck-
ered.
Case 3; Mary Dum?, aged 38 years,
had experienced severe moral afflictions,
and entered the Cochin Hospital, on the
5th September. She had been affected
with jaundice for eight months previously,
whieh she attributed to mental anxiety.
After a miscarriage in the month of June
preceding, a tumour slowly formed in the
right sideof the abdomen,unaccompanied
? by any pain. On examination at the hos-
pital, this tumour was of considerable
dimensions, being moveable, and appa-
rently the size of a foetus, rather attached
to the right hypochondrium. There was
fluctuation in the abdomen, but no oedema
of the lower extremities. The patient
complained of great debility, but not of
any pain. The skin nnd eyes were yel-
low, inclining to a green hue. The urine
was of the same colour. The stools were
. generally white?sometimes black and
watery. M. Cayol and others examined
. the patient, but were unable to determine
on the nature of the disease. The pa-
tient died on the 25th September, in ex-
treme marasmus.
Dissection. The abdominal cavity con-
tained a large quantity of yellow serum.
-The liver was of middling size, hard, and
-apparently infiltrated with yellow bile.
On its inferior surface were three large
tubercles. The gall-bladder was enor-
mously distended, being the size of a
child's head. It touched the spine be-
hind, and bulged out the abdominal pa-
rietes in front. The neck of the gall-
bladder, as well as the ducts, were en-
circled by a mass of tubercles extremely
hard, in which was involved the head of
the pancreas. The duodenum was com-
pressed by this mass against the spine,
but: not obliterated. The immense gall-
bladder was filled with a dark-coloured
viscid fluid, and contained more than a
hundred biliary calculi, of various sizes.
The parietes of the gall-bladder were very
much thickened. The stomach was enor-
mously distended with half-digested ali-
ment. There was no other disease of
importance.
This case will elucidate some observa-
tions which we made in a preceding num-
ber?especially 011 a case that occurred
in Panton Square.
Case 4. Obliteration of the Cystic Duct.
A female was brought to the hospital for
the treatment of acute pneumonia, of
which she died, in spite of all the means
they could use. She was evidently jaun-
diced?and it wasremarkedthat after each
bleeding, for the pneumonia, the colour
became deeper. On dissection, the gall-
bladder was found much distended with
bile, and its duct completely obliterated.
There was inflammation of the stomach,
and ulceration of the pylorus. Bile was
found in the small intestines. It was
interesting to observe, in this case, that
after each bleeding, the yellow colour of
the skin and eyes became more and more
intense.
Several other cases are detailed by our
author, which we cannot notice in this
paper. In one case there was found a
great dilatation of the ductus communis
?ductus cysticus?and ductus hepaticus,
without any ostensible obstruction in any
of these canals. But there was inflam-
mation of the mucous membrane of the
duodenum and small intestines, which
-was probably the cause of the dilatation,
by preventing the bile from getting into
the primse vise.
The author enters his protest against
the use or rather the abuse of irritating
and often repeated drastic purgative me-
dicines which, he thinks, produce in-
flammation of the excretory tubes of the
liver?gall-stones?and even hepatitis.?
Journal. Complementaire.
1828j Mu. Christian on Ophthalmia Porriginosa. '531
3. LIVERPOOL OPHTHALMIC
INFIRMARY.*
Ophthalmia Porriginosa.
Under this term, Mr. Christian, consult-
ing surgeon to the Liverpool Ophthalmic
Infirmary, has treated of an affection
which is usually denominated scrofulous
ophthalmia. Mr. C. thinks himself au-
thorised to give the disease this title, not
only from its appearing in connexion with
porrigo, but from its bearing certain fea-
tures, sufficiently characteristic in them-
selves, to warrant such a distinction.
The coincidence has been regarded as
casual, and the ophthalmic disease treated
on general principles. Not unfrequently,
when the symptoms have run high, the
porriginous eruption has beenencouraged,
with the hope of relieving the local affec-
tion. As Mr. Christian looks upon these
two in the relation of cause and effect,
he conceives that treatment of derivation,
according to that principle, must be er-
roneous, if not injurious. The following
description of the disease is worthy of
record in this place.
" Porriginous ophthalmia is a disease
of early life, affecting principally children,
though sometimes seen in the adult sub-
ject. It is usually accompanied by an
eruption of pustules on the face or head,
which go through the various stages of
suppuration, ulceration, and desquama-
tion : and if the eruption in its pustular
form shall have disappeared before the
inflammation of the eyes have commenced,
still there will, almost always, be found
some traces of the original disease, ia
the form either of scabs or fissures, situ-
ated behind the ears, at the commissures
of the palpebrae, or at the junction of the
alas nasi with the cheeks. It is worthy
of remark, that when the fissure or chap
is situated between the lips, attended
with excoriation of the nostrils, the upper
lip often swells, assuming the appearance
of what is vulgarly termed the scrofulous
lip, whicli may be one cause of the dis-
ease being referred to this origin.
" Sooner or later, however, the oph-
thalmia commences, and the eye in a
short time presents a highly vascular
state of the conjunctival membrane. The
inflammation appears in different degrees
of intensity, in different parts of the albu-
ginea; and the vessels, which are. much
enlarged, are seen to run in clusters, to-
wards certain parts of the cornea, whilst
this transparent tunic contiguous to these
vessels is more or less clouded. On mi-
nute inspection, a pustule or vesicle will
frequently be discovered at the apex of
each of these fasciculi, or bundles of red
vessels; but, very often, depressions will
be found to exist, instead of raised pus-
tules, situated at the margin of the cornea,
or on the intermediate surface. On some
occasions, the cornea will be perfectly
transparent, and free from either pustule
or ulcer, whilst the albuginea, with its
vessels fully distended with blood, will
present one or more yellowish spots, ap-
parently elevated above the surrounding
vascular superficies. These are so many
ulcers, which by their extreme irritability
keep up, if not give rise to, the inflam-
matory excitement in these parts. Simi-
lar ulcers are sometimes found on the
lining membrane of the palpebra;. Wher-
ever they are situated, they render the
motions of the eyelids very painful; so
that the eyes are generally kept fast
closed, and their inspection, in conse-
quencei becomes a matter of extreme
difficulty. The sight being affected, only
in proportion to the degree of opacity,
and as this is but inconsiderable in some
instances, the vision remains perfect;
but the sight will necessarily be more or
less impaired, according to the extent
and density of the opacity of the cornea.
Very often black spots may be observed
arising from the attenuation of the corneji,
occasioned by the ulcerative process,
which sometimes perforates this tunic,
and causes the incarceration of a portion
of the iris. The discharge which issues
from the eye, consists principally of tears
mixed more or less with a sanious fluid
which discolours the linen applied to the
parts, and is often considerable in quan-
tity. Although the pain attendant upon
this affection of the eyes is not very great,
yet, from the great irritability of these
parts, the patient not only carefully shuns
the light, but desires to lie with the face
downwards, whilst the hands are almost
unceasingly applied to the forehead."
The disease often assumes a chronic
form, and lasts for months, leaving one
or more opake spots on the cornea, the
consequence of effused lymph. The con-
Glasgow Medical Journal, No. 1.
3 M2
532 Periscope; or, Circumspective Review. [March%
junctivapalpebralis also becomes changed
in structure, and a morbid sensibility is
generated, which is very difficult of re-
moval. In such cases the disease will
continue long after its cause is removed,
and then requires a specific treatment.
The author next goes on to prove, or at
least to support his doctrine, that the
affection of the eye is an association with,
or consequence of, the cutaneous erup-
tion. For the arguments and observations
on this head we must refer to the original
paper. His principle being admitted,
(and we do not see any just cause for
denying it) the indications of cure will
hinge on the removal of the eruption, and
the extinction of the ulcerative process.
" Whatever external appearance of
active inflammation may exist, provided
it be of the specific character, usually at-
tendant on porrigo, and occurring in con-
junction with pustules or ulcers of the
" globe, bleeding either locally or generally
is seldom necessary ; except, indeed, the
inflammation shall have extended to the
internal tunics of the eye characterized
by pyrexia, severe pain of the eye, fore-
head, &c. Blistering, likewise, which
constitutes so valuable a remedy where
a derivative is required in these cases, is
not only useless, but generally tends to
aggravate the eruptive disease, and there-
by proves an additional source of irrita-
tion. The cooling sedative lotions in such
general use are for the most part una-
vailing here : the object kept in view in
the treatment of this particular affection,
being not so much the alleviation of pain
?? and irritation, as the production of a new
action, in parts already under the influ-
ence of a specific disease.
" Corresponding with these views, it
has been found by experience, conducted
upon rather an extensive scale, in a pub-
lic institution, where a large proportion
of the cases are of this class, that the
mercurial applications constitute the best
remedies. A weak solution of the oxy-
muriate of mercury, composed of one
quarter of a grain to the ounce of water,
forms a very useful application : or if
there be much discharge from the eyelids,
? and especially if accompanied by excori-
ation of the parts around the eye, the
mixture of calomel and lime water, known
by the name of black-wash, will be found
to be one of the best local remedies. The
unguentum hydrargyri nitratis mitius,
or the unguentum hydrargyri praecipitati
albi, affords an excellent dressing for the
eruption, or ulcers about the face and
ears, which require to be attended to ;
a small portion of the former, or the red
precipitate ointment diluted, is to be in-
troduced within the palpebrse at bed-
time.
" Previously, however, to the use of
these latter means, a weak solution of
the argentum nitratum, in the proportion
of two grains to the ounce of distilled
water, should be dropt on the surface of
the globe, and.this ought to be repeated
every second day, as long as the ulcers
continued.* It has already been re-
marked, that the morbid action has been
kept up, in the fine textures of the eye
by the presence of an ulcer, or fissure at
the corner of the eyelids, which will be
frequentlyobserved tobleed, whenever the
palpebrae are forcibly separated. These
fissures must be touched, every second
day, with a saturated solution of the
nitrate of silver; and during the inter-
mediate days, with the weak ointment
of nitrate of mercury.
" The vascularity together with the
irritability will generally disappear with
the healing of the ulcers, a strong proof
that the specific action of these vessels is
overcome; but should a degree of mor-
bid sensibility still remain, a collyrium
consisting of four grains of the sulphate
of zinc, to an equal number of ounces of
water, combined with a drachm of the
vinum opii, dropped into the eye, once
or twice a day, will be found highly
useful."
On the constitutional treatment, Dr.
Willan's work will be consulted with ad-
vantage, and also the lectures of Mr.
Lawrence, as published in the 10th volume
of the Lancet.
* We are informed by Dr. Maule, of
Marlborough, that " he has found a so-
lution of the argentum nitrat. in the pro-
portion of two grains to the ounce of
distilled water, (dropt into the eye twice
a day), a safe, not painful, and very
powerful remedy in inflammation of the
conjunctiva."?Extract from a Letter to
the Editor.
1828J ? Gangrene of the Compound Fracture. 533
.3. ST, GEORGE'S HOSPITAL.
Compound Fracture of the Thigh,
TERMINATING IN MORTIFICATION.*
The patient, a stout muscular man, was
admitted under the care of Mr. Rose,
with compound fracture of the left thigh,
about two inches above the knee-joint.
The accident had happened half an hour
previously, in consequence of a large
wool-sack falling on his shoulders, and
felling him to the ground, with the leg
bent under him. The fracture was trans-
verse, the wound about an inch and a
half in breadth in the same direction,
but the fracture seemed to extend down
through the inner condyle, where a pro-
jection of bone was felt beneath the in-
tegument. There had been much haemor-
rhage, and the disposition to bleed still
continued. Limb to be placed on the
double inclined plane?light compress
and dressing to the wound?cold lotion,
with occasionally ice to the knee?ano-
dyne draught. On the next day, there
was a slight attempt at reaction. V. S.
ad ?viij. The limb became much swollen
from extravasation?there was thirst, and,
in the evening, the pulse had got up to
110, with heat and tension of the integu-
ments of the thigh and knee. Haust.
sennas?V. S. ad ? viij.
Third day. Better in the morning, hav-
ing passed a quiet night, but at 10, p. m.
he became rather delirious, and an em-
physematous crackling could be felt on
pressing about the thigh. Liq. op. sed.
ni. xxx. Spt ceth. comp. Tl\. xxx. Mist,
camph. ?j. statim. Fourth day. Pain had
ceased?the leg and foot were cold?the
pulse weak ; in short, mortification had
shewn itself. This spread to the groin,
the emphysema reaching still higher?
the skin assumed a bilious tinge?the
countenance became puffy and anxious,
he grew semi-delirious, and at 2, a. m. of
the sixth day, died.
The dissection we shall give in the
words of our contemporary.
" Sectio Cadaveris, 12 hours after death.
?On uncovering the body it presented,
certainly, a most extraordinary appear-
ance. It was blown up to at least twice -
its natural size, and emphysematous from
top to toe, whilst even the features were
so distorted, that the nearest friends of
the patient could scarcely have recog-
nised him. The mortification had ex-
tended for some little distance up the
abdomen on the left side ; the scrotum
was like a large green ball; and the
penis was discoloured, and in a state of
priapism. No attempt at union had
taken place in the wound, and on cutting
down to the fractured bone it was found
to be much shattered. As has been
stated, the femur was broken across
about an inch and a half above the knee-
joint, but from this a perpendicular frac-
ture extended into the joint separating
the inner condyle. Splinters of bone
were found here and there; the cancelli
were gorged with blood, which had also
been forced into the vasti, crurseus, and
other muscles, but more especially into
the parts around the knee-joint, and
within it. On cutting into the capsule
of the latter, there flowed out a most
offensive mixture of grumous blood and
sanies, and the cartilaginous surface of
the patella and of the condyles of the
femur was stained of a dark venous co-
lour, to the depth of a line or more.
No injury of the femoral or popliteal ves-
sels could be discovered, but from the
quantity and situation of the extravasated
blood, it appeared probable that some of
the articular, or the anastomotica magna
artery, or both, had been torn. The em-
physema was found to be seated not so
much in the subcutaneous cellular tissue,
as in that looser texture which connects
and pervades the muscles, &c. It was
surprising how superficial the gangrenous
disorganization appeared to be above the
immediate seat of injury. The cellular
texture and the muscles were emphyse-
matous to be sure, but neither the one
nor the other showed any trace of disease
besides. The muscles indeed were as
florid, and seemingly as healthy, as an
anatomist could desire. Nothing par-
ticular, we believe, was found in the ab-
domen or in the thorax. The head was
not examined."
Remarks. The above is a good instance
of the traumatic gangrene of Baron
Larrey, or " local gangrene," described
by Mr. Guthrie in his very excellent work
upon gun-shot wounds. This form of
gangrene, it is well known, may arise
from two causes, 1st, defect of power in
* London Med. Gazette, No. 11.
534 Periscope; oh, Circumspective Review. [March 8
the limb from wound of the great vessels
which supply it, or destruction of its tex-
tures ; and, 2dly, from excess of inflam-
matory action kindled up after slighter
injuries. Mr. Guthrie, in his work cited
above, mentions two or three cases of
the first species, in which he amputated
with success, no line of separation having
formed. Sir Astley Cooper, if we re-
member aright, details two cases of trau-
matic gangrene, in which amputation,
under the same circumstances, was suc-
cessfully performed, and strongly recom-
mends the practice. A year or two ago
a case occurred to Mr. Brodie at St.
George's Hospital, where compound frac-
ture of the bones of the leg, and that not
a very severe one, was followed by mor-
tification of the limb. Mr. B. amputated
the thigh, without waiting for any line of
separation, but unfortunately tetanus su-
pervened and carried off the patient.*
In the present case, it appears that am-
putation was proposed to the patient at
the time of his admission, but he refused
to submit to it. It was not again pro-
posed, says the reporter, upon the first
appearance of the mortification, first, be-
cause it was thought that he would not
consent, and, secondly, " because the
chance of success was then necessarily
desperate."
Now, really, we do not conceive
these reasons of the reporter's to be
particularly good ones. As for thinking
that the patient woidd not submit to
it, that was neither here nor there, for a
few minutes' conversation with him would
have settled the point. A man may re-
fuse to submit to an operation in the first
instance, because he is not aware of the
extent of the injury; but when he finds
his limb turning black and blue, in other
words, mortifying, he may become alarm-
ed, and refuse consent no longer. Again,
the chances of success, we are told, were
" necessarily desperate," but all we can
say is, that there are several cases upon re-
cord where amputation saved the patient.
The chances are not very great, to be sure,
but still we do not see why they should
be " necessarily" desperate.
4. STRASBURGH LYING-IN HOS-
PITAL.
Humoral Pathology.
The following curious case is reported by
M. Stoltz, from the Strasburgh Lying-in
Hospital. A female, aged 20, was ad-
mitted into the Clinique, on the 28th
May, 1826, being then in the eighth month
of her second pregnancy. On the 23d
July, pains of an intermitting character
came on in the loins, and were considered
parturient. But no dilatation of the os
uteri took place, and, on the 15th, she
was still in pain. 16th. Febrile symp-
toms set in, and, in the evening, red spots
appeared on various parts of the body,
some of them being elevated, and giving
rise to the suspicion that they were of the
varioloid character. 11th. A good deal
of blood came away in the urine, and a
petechial eruption was evident over the
members, trunk, and inside of the mouth,
even on the tongue. The breathing was
slow and laborious?there was a sense of
constriction in the throat?febrile heat of
skin?small, quick pulse?vertigo?great
debility. These symptoms increased, and
she died next day delirious. At this mo-
ment, M. Stoltz was called in, and al-
though no motion of the foetus had been
observed since the 16th, he determined
on the Caesarean operation. But the
child was evidently dead some days, for
the epidermis readily detached itself from
all parts of the body. Over the surface
of the mother, the same petechial spots
still existed, and were seen on the tongue,
and the conjunctiva of the eye. There
was nothing particular in the head. The
lungs were covered with petechise, and
much blood and mucus flowed from in-
cisions into their structure. The pleura,
the surface of the heart, the exterior of
the large vessels, the peritoneal covering
of the stomach, intestines, spleen, .and
kidneys, all presented petechial ecchy-
moses. The internal surface, also, of the
pelves of the kidneys shewed petechiae,
and a quantity of blood. On the surface
of the foetus, there were no petechiae;
but the lungs were covered with them,
as were the pericardium, heart, and ori-
gins of the large vessels. The blood, both
in the mother and fetus, was entirely li->
quid, and of a violet colour. There were
no coagula in any part of the venous or
arterial systems of either of the bodies.
* This case was published by us at the
time, and may be found fully detailed at
page 219 of our 13th number.
1828] Inflammation and Softening of the Medulla Spinalis. 535
We think there can be little doubt that,
in this case, the physical phenomena pre-
sented by the foetus proved the identity
of disease in the mother and child. Pe-
techia; were found in both bodies?-and,
in both, the blood was in the same dis-
solved state. After this, can anyone doubt
the communication between mother and
foetus?although no injections can be
made to pass?no nerves can be traced ?
5. HOPITAL DE MONTPELLIERi
M. Boyer and M. Delpech on Pili-
J1ICTION.
We lately noticed some curious instances
of " bodies foreign in bodies natural," as
adduced by Messrs. Brodie and Bell. Un-
der the somewhat eccentric term of " Pi-
limiction" (the meaning of which is ob-
vious enough) M. Boyer has bi-ought for-
ward some remarkable examples, in the
Royal Academy of Medicine.
Case I. A female, (the wife, we pre-
sume, of a medical man) at the age of 25,
and in her second pregnancy, became af-
fected with irritation in the bladder, and
voided frequently some hairs with her
urine (pilimiction.) After some time, her
husband (in the presence of M. Delpech)
extracted from the patient's bladder, by
means of an instrument, a mass of hair,
of considerable size. Several other ex-
tractions were subsequently performed.
But, at length, she came into the hospital
of Montpellier, and M. Delpech, having
first dilated the urethra, was enabled to
reach the bladder with his linger, and ex-
tract a large ball of hair. The patient
continued well for two years, at which
period, she evinced symptoms of stone in
the bladder, and M. Delpech removed a
calculus, the size of an egg, the nucleus
of which was a piece of skin, covered with
hair, and containing a portion of zigo-*
matic bone ! M. Delpech explains this
case, by supposing that the debris of an
imperfect foetus, had made its way through
the parietes of the uterus or ovaria into
the bladder; and, indeed, we cannot see
any other rational explanation of the phe-
nomenon.
Case 2. By M. La Riviere. A lady,
58 years of age, had complained, for se-
veral years, of a sense of weight in the
lower part of the abdomen, with difficult
micturition, and great pain in the region
of the bladder. M. Gille, surgeon of the
Hotel Dieu, sounded her, and struck upon
a tumour, which burst, and discharged a
large quantity of matter. The pus con-
tinued to flow, in considerable quantities,
with the urine, for eight days, when fe-
ver supervened, with diarrhoea, vomiting,
and sweats. The case ended fatally. On
dissection, the uterus was found adherent
to the bladder, and, in this adherent por-
tion, were found several pieces of bones,
and also some hairs, in a kind of cyst;
from which the pus had issued into the
bladder.
The celebrated Meckel, indeed, has
collected, in an interesting memoir, the
most remarkable examples of masses of
hair found in different parts of the human
body. He quotes from Schenk, Horstius,
Hildanus, Tulpius, and a multitude of
others, various cases of masses of hair
passed from the bladder, but no dissec-
tions have proved that they originated in
that receptacle. We have no doubt that
they proceed, in all cases,from the uterus,
the fallopian tubes, or the intestinal canal.
6. LA CHARITE.
Inflammation and Softening of the
Medulla Spinalis.
[M. Bayle. La Charite.]
The following very interesting case will
be found to illustrate and confirm some
important physiological and pathological
discoveries of recent date.
Case. Agustus Barre, aged 20 years,
by trade a shoemaker, ricketty from his
infancy, but having enjoyed good healthy
became affected, about the middle of
December, 1825, with pain in the left
side of his neck, which he attributed to
cold, and to which he paid little attention
for several months, when it gradually in-
creased, and at length impeded him in
his trade. At this time also Barre began
to feel a weakness, accompanied with
flying pains in his arms. He had leeches
applied to his neck, took vapour baths,
and some medicines, with slight ame-
lioration of the symptoms, On the 15th
536 Periscope; on, Circumspective Review. [March 8
April, 1826, he was received into La
Charit6, complaining of the pain in his
neck, which prevented him from turning
his head, and also of pain and weakness
of the left arm. The complaint was con-
sidered rheumatic, and leeches, blisters,
low diet, and some topical applications
were employed, with trifling effects. He
now complained of pain in Jiis head, while
that in his neck became aggravated. On
examination, a diffused swelling was ob-
served on the left side of the neck, which
was treated unsuccessfully by liniments
and other outward applications. Towards
the latter end of May, the cervical pain
had much increased, and evidently ex-
tended itself to the head, especially to.
the frontal region. The left arm first,
and subsequently the right became gra-
dually paralytic, and by the end of June
they were both completely incapable of
motion, while their sensibility was in a
state of perfect integrity. The left lower
extremity came into the same state, and
soon after the right leg lost its muscular
power, retaining its sensibility. The in-
tellectual faculties were yet untouched ;
but the speech was embarrassed, and the
patient complained of difficulty of breath-
ing, and a feeling as if the chest were
compressed. The respiratory murmur
was heard throughout the thorax, but
louder in the right side. The patient
now emaciated fast, and there was occa-
sionally an acceleration of the pulse.
During the first four days of July, he
evinced incoherency of ideas, with com-
plete delirium at night. He died on the
4th July.
Dissection, 36 hours after death. The
tumour on the left side of the neck was
first examined.. It was rather firm, and
appeared to have a deep-seated base.
After removing the integuments, and
raising the sterno-cleido muscle and
pneumo-gastric nerve, an abscess was
discovered opposite the transverse pro-
cesses of the cervical vertebrae, the size
of an egg, containing yellow and thick
matter. When a probe was passed into
the abscess, it penetrated into the spinal
canal. The vertebral column was then laid
open throughout its whole .extent. The
posterior columns of the medulla spinalis
were found softened to the consistence
of cream, from the first cervical vertebra
to the third or fourth dorsal. This
softening or diffluejicc was greatest at
the surface, and gradually diminished as
the centre of the chord was approached.
The anterior portion of the spinal marrow
was slightly softened also, but infinitely,
less so than the posterior. About the
middle of the cervical region, two small
holes were found in the bodies of the
vertebrae, leading to the external abscess.
The transverse processes were here ca-
rious. In the head, the arachnoid was
found greatly injected, and adherent in
some places to the pia mater, as was the
latter to the substance of the brain. The
brain itself and cerebellum were sound.
The right lung was gorged with blood,
especially posteriorly, where it was also
liepatized, and there were numerous tu-
bercles scattered through its substance.
The left lung was more crepitous and
free from disease. On opening the ab-.
domen, they were surprized to find four,
perforations of the stomach?some of
them an inch in diameter. Near these
perforations the mucous membrane of the
stomach was thinned away or entirely,
obliterated.
Remarks. M. Martinet thinks that the:
external swelling and abscess were only
consequences or prolongations of inflam-
mation of the spinal marrow?and, in
fact, that the matter had made its way
outwards through the holes in the carious,
vertebrae. This, he thinks, is proved by
the fact that, from the very beginning,,
the patient complained of weakness and
flying pains in his arms. We confess we
have some doubts whether the matter of
the abscess made its way inwards or
outwards. The extension of inflammation
from the intermuscular swelling to the
interior of the canal (a thing not unlikely)
might occasion the early symptoms above-
mentioned. Nay, might not the swelling
on the neck affect the nerves issuing from
the spinal column, and produce the early
phenomena ? Be this as it may, there
can be no doubtof the existence of inflam-
mation of the medulla spinalis, whether
primary or secondary?and of the soften-
ing or diffluence being a consequence
of this inflammation. We observe that,
as soon as this disorganizing process had
made a certain progress, the upper and
one of the lower extremities were stricken
completely paralytic,their sensibility, how-
ever, Remaining entire?a pathological
fact which beautifully illustrates and
1828] Extirpation of a Sarcocele, followed by Tetanus. 537
confirms the experiments of Bell and
Magendie ; for it was seen that the pos-
terior portions or columns were disor-
ganized, from which portions the nerves
of motion arise. The delirium which
supervened during the last four days of
the patient's life, left in the membranes
?f the brain the proper signs of its
existence?or rather the cause of the
phenomenon. The perforations of the
stomach are not so easily accounted for.
Were they ante-mortem or post-mortem
disorganizations ? It will probably be
said that these lesions and destructions
?f the coats of the stomach could not
have taken place during life, otherwise
they would have been attended with cor-
responding symptoms. But it is to be
recollected, that the delirious state of the
patient, for four days, might possibly
mask the affection going on in the sto-
mach. The explanation of this remark-
able phenomenon, indeed, is not very
easy on any hypothesis.?Bevue Medi-
care.
The above case, we think, will bear
upon and help to illustrate the case lately
published by Mr. Iliff, and noticed at
page 192 of this Number. We imagine
that it will hardly be contended, after
this, that Mr. Iliff's patient laboured
under no inflammation of the spinal
marrow anterior to the diffluence of that
organ, and the complete loss of all its
functions.
Before we quit this subject, we may be
permitted to notice another curious case,
related by M. Martinet, in the same
hospital report. A young gentleman had
experienced, for twelve or fifteen days,
an intense head-ache, principally at the
bottoms of the orbits, and then was
seized with delirium, fever, agitation and
occasional vomitings. He was twice bled,
and blisters were applied to the feet. On
the fourth day from this accession, the
patient appeared to be fast approaching
the final goal, and had the rattles in his
throat. In this state eight grains of
emetic tartar were administered. From
that moment the patient's agonies seemed
inexpressible, and he died the next day.
Dissection. The internal surface of
the dura mater was found, in some places
covered with a layer of nearly fluid pus?
the membrane itself was highly injected.
A great portion of the superior surface
of the hemispheres presented the ap-
pearance of pus, being soft, yellow, and
nearly liquid. The same was seen in the
fissura magna, and several other parts of
the brain's superficies. The pia mater
was every where intensely injected. On
opening the abdomen, the stomach was
found perforated in two places, and a
quantity of brownish lluid extravasated.
One of the openings was an inch in dia-
meter. Around these openings, the pa-
rietes of the stomach were very much
attenuated, and somewhat reddened.
But the general surface of the stomach
was pale. All the other organs were
sound.
This last case shews to what a pitch of
disorganization even the brain will some-
times go, without corresponding symptoms
during life. When we say corresponding
symptoms, we mean as to duration. We
can hardly wonder at the perforations in
the stomach, after a dose of eight grains
of tartar emetic administered to a patient
in articulo mortis ! Had the physician
applied this medicine to his own skin, he
would soon have found out the irritating
qualities of the drug.
7. HOSPICE GENERAL. .
Extirpation of a " Sarcocele," fol-
lowed by Tetanus.*
Tetanus has, we believe, occasionally,
though very rarely, followed amputation
of the testicle, but in such instances it
has generally been referred to the sur-
geon's having comprised the cord, vessels,
nerves, and all, in one ligature. In the
case we are going to detail, this was not
done, but other violence was inflicted on
the cord which would seem to account
for the tetanic symptoms.
Case. Muyart, set. 37, who had led a
drunken, dissipated life, and suffered
from venereal complaints, receivedablow
on the testicles in the beginning of 1823.
No swelling ensued, but in January,
1825, he applied at the Hospice General,
(of Rouen, we believe) suffering from con-
siderable pain with some swelling, and
induration of the left testis. In spite of
the means employed (no mercury was
* M. Couronnc. Revue M^dicale,
Sept. 182/;
538 Periscope ; on, Circumspective Review. [March 8
used) the swelling progressively increas-
ed, whilst the pain, pari passu, diminish-
ed. Towards the commencement of July,
M. Couronne conceiving the case to be
one of sarcocele, and not likely to he be-
nefited, proposed the operation to the
patient to which he readily consented.
A moderate incision was first made into
the tunica vaginalis to be sure that it was
not a hydro or hematocele, but not a
drop of fiuid was found in its cavity.
The removal of the testicle was then com-
pleted in the usual way, but, on dividing
the cord, the upper portion retracted
within the inguinal canal. The retrac-
tion, however, was not permanent, for
alternately it appeared and disappeared,
evidently depending on the irregular
contraction of the cremaster muscle. In
order to tie the vessels, it was necessary
to seize the end of the cord by the for-
ceps and draw it out, which occasioned
very severe pain running up to the loins
cf the same side.
On examination of the tumour, it was
found to be of a mixed character, some
portions resembling scirrhus, others the
medullary sarcoma, with, here and there,
small cysts containing fluid as in what is
commonly, though not very correctly,
called " hydatid disease of the testicle."
A good deal of pain remained for some
time after the operation, but, in the
course of a day or two, it subsided, ap-
parently in consequence of a certain de-
gree of bleeding which had taken place.
On the 10th day, there came on some
stiffness of the lower jaw, and on the
12th, there was decided trismus. Com-
plete tetanus followed, which was treated
by bleeding, laudanum, the warm bath,
mercurial frictions, &c. and, on the 16th
day, he was a good deal relieved. This
was the 29th of July, but on the 30th,
a change for the worse took place; the
jaws were permanently locked?the mus-
cles of the abdomen, arms, and neck, ri-
gidly contracted?deglutition almost im-
possible?great anxiety, pain in the loins,
and insomnium. Convulsions, and loss
of consciousness came on, and, on the
3rd of August, the patient died. The
wound during this time, so far from tak-
ing on an unhealthy appearance, had
gone on rapidly cicatrizing.
Sectio cadaveris. There was some em-
physema beneath the skin, and amongst
the muscles of the back and neck. The
substance of the brain was a little in-
jected, and a trifling quantity of bloody
serum was found in the ventricles. The
tunica arachnoides of the cerebellum was
quite opaque, and the substance of the
cerebellum itself softened throughout,
and reduced at its circumference to a
semi-fluid bouillie, of a yellowish grey'
colour. The medulla oblongata was un-
affected, but the spinal marrow, in its
upper half, presented strias, and patches
of red in its substance. The spinal du-
ra mater, was much reddened on its in-
ner surface, and between it and the bony
canal there was discovered a considerable
quantity of bloody serum. In the left
side of the thorax there was some effu-
sion, and the lower lobes of the lung
were exceedingly small and filled with
dark coloured blood.
We suppose, for we know of nothing
better that can be offered, that the cause
of the tetanus in this case, was the vio-
lence done to the cord by dragging it
out, which produced, at the time, and
indeed for a day or two afterwards, very
considerable pain. This may have been
the cause, or it may not, for the truth is
that we know little or nothing of either
the etiology, pathology, or treatment
of these terrible convulsions of the ner-
vous system. The cicatrization of the
wound during the progress of the teta-
nic symptoms is a curious but not very
uncommon circumstance, and it proves
that these latter are not dependent, as
some would have us believe, merely on a
kind of error loci.
8. GLASGOW INFIRMARY.
Cases of extensive Burn, tkeateju
with Cotton.*
Case 1. John Cunningham, mt. 25,
* Surgical Report from the Royal In-
firmary of Glasgow. By I. H. Plymsoll,
Esq.*
* Having accidentally learnt that a se-
vere burn in this metropolis had been
very successfully treated, by a methodlittle
known in this country, though employed a
good deal in America, we have much plea-
sure in laying before our readers some
authentic facts relative to this mode of
treatment, from the Royal Infirmary of
Glasgow.?Ed.
1828] Treatment ok Burns by Cottons 539
labourer, was admitted into the Glasgow
Koyal Infirmary, March 3d, 1827, under
the care of Dr. Anderson, in consequence
?f a burn which he had received, a few
hours previously, from the explosion of
gas in a COal-pit. Face and upper extre-
mities were burned to a great extent, and
lrJ a state of complete vesication. Sweet-
?jl was applied immediately after the ac-
cident :?Injured parts to be dressed with
finely-carded cotton. 12th. Cotton has
teen removed, and burned surface looks
healthy, and partially cicatrized. Has
been frequently burned before, but never
felt so easy as under the present treat-
ment : previous burns caused great dis-
figuration of countenance, which has been
entirely obliterated under the application
of the cotton. April 4th. Burned surface
completely cicatrized?health good. Dis-
cussed, cured.
Case 2. Alexander M'Kinlay, ret. 21,
labourer, was admitted into the Infirmary,
April 16th, 1827, under the care of Dr.
Anderson, in consequence of a burn which
had been inflicted, as in the former case.
Pace, right arm, and middle third of legs,
are vesicated : sweet oil was applied im-
mediately after the accident. Complains
of severe pain on injured surface. Injured
parts to be dressed with cotton-wool.
Has been much easier since the cotton
Was applied. 26th. Continues free of
uneasiness, and sleeps well. June 12tli.
Burned surface entirely cicatrized, and
without contractions. Cured.
Case 3. John Nudgent, aet. 23, la-
bourer, was admitted into the Infirmary,
Nov. 22d, 1827, under the care of Dr.
Anderson, with an extensive burn, which
he had received, three days previously,
in consequence of his clothes having caught
fire. Almost the whole extent of back,
right side of abdomen, right and left
thighs, knees and upper part of legs, and
left elbow, are most severely burned.
Burned surface is covered with a leathery
slough, excepting here and there, and on
right thigh, where there are vesications.
Around the edges of slough skin is red, and,
in some places, vesicated. The sloughing
parts were bathed with the spirits of tur-
pentine, and, afterwards, covered with cot-
ton wool and bandages. 23d. Has been
easier since the cotton was applied than
at any time since the receipt of the injury.
The sloughs were altogether separated
after the third or fourth dressing, and
expose a healthy granulating surface, gra-
nulations having afterwards become ra-
ther prominent. Patent lint, soaked in
a solution of the chloruret of lime, and
covered with sheet lead, was afterwards
applied. This had the effect of bringing
the granulations almost on a level with
the adjoining skin; but new skin suffered
from pressure?the patent lint and lead
were, therefore, discontinued, and cotton
was again applied, with increased pres-
sure. From this time, he progressively
recovered. New skin continued to form
rapidly. Has been free of uneasiness, and
slept well every night, since admission.
Injured surface is now almost completely
cicatrized.
Case 4. Daniel Lochrie, aet. 50, la-
bourer, was admitted, Nov. 25, 1827,
under Dr. Cooper, having, a few hours
since, fallen into a vessel containing a
quantity of boiling soda ley. Head and
face, and superior part of thorax and up-
per extremities, were burned. Injured
parts are vesicated, and extremely painful
?eyelids are swollen, and conjunctiva
inflamed :?Dressed with cotton-wool.
Dec. 1?<. Cotton has been removed from
left arm, on account of discharge?parts
were found doing well. Jan. 20tli. In-
jured surface cicatrized through its whole
extent.
Observations. These cases are remark-
ably illustrative of the advantageous
consequences resulting from the applica-
tion of cotton to burns, and, in conjunction
with other cases, which have been treated,
of late, in this infirmary, almost equally
severe, and terminating equally success-
ful, have considerably influenced the de-
termination of the surgeons to this hos-
pital, in the treatment of these injuries,
so that the practice has, ultimately, be-
come established. The application of
cotton to burns originated in America,
where, I understand, it has been practised
exclusively in superficial burns. It has
been introduced in this country by Dr.
Anderson, of Glasgow, who has consti-
tuted it a most important innovation in
the treatment of these injuries. Dr. An-
derson has practised it extensively, both
in superficial and deep-seated burns; and,
convinced of .its decided superiority ovev
540 Periscope ; or, Circumspective Review. [March 8
every other method of treatment, and of
the beneficial consequences which would
result from its more extensive application,
has strenuously recommended this prac-
tice to his professional brethren, and has
been indefatigable in demonstrating the
beneficial results of its operation?exhi-
biting a strong determination that the
practice should become more generally
established. Cotton cannot be considered
to have any specific influence on burns
any other substance, of a similar nature
and consistence, would have the same ef-
fect. It constitutes, from its peculiar
softness of texture, and unirritating qua-
lities, a soothing and comfortable appli-
cation, which absorbs the discharge from
the wound, and forms with it a case, sub-
stituting the place of the original cutis,
and affords a thorough protection from
the approach of the external air, which
would exert a very deleterious effect, if
allowed to come in contact with the in-
flamed or raw surface occasioned by the
burn. It is a non-conductor of heat, and,
consequently, diminishes that excessive
expenditure of heat, which would other-
wise be evolved from the injured surface,
and which is productive of such injurious
consequences. It has the power of ac-
commodating itself more than the ordi-
nary applications, to those irregularities
of surface which are generally occasioned
by extensively-deep burns, and, conse-
quently, of being brought into more close
approximation with the injured parts.
Bandages can be applied with almost any
degree of pressure, without producing
irritation ; an equability of pressure is,
therefore, kept up on the whole extent of
the injury, which has the effect of level-
ling irregularities, and of repressing the
growth of exuberant granulations, and,
\iltimately, of effecting a regular and un-
contracted surface. The skin which is
formed under this treatment is not a
smooth, continued skin, as in other cases,
but possesses almost the same degree of
mobility and elasticity, and those insen-
sible indentations, which are observed in
the original skin. This method of treat-
ment, therefore, is particularly advanta-
geous in burns where the joints are in-
volved, and also in burns of the face, and
other parts which are exposed to view, as
a cure is effected without restraining the
tnotions of the joint, and without exhi-
biting the slightest deformity of the face
?this is well exemplified in the case
of Cunningham. The granulations will
sometimes become prominent, in conse-
quence of a sufficient degree of pressure
not having been kept up. In order to
obviate this inconvenience, patent lint,
covered with sheet lead, may sometimes
be substituted with advantage. Should
this produce much irritation however,
cotton, moistened either with a solution
of the sulphate of copper, or chloride of
lime, should be again applied, and with
increased pressure: this will have the
same effect, without being productive of
any uneasiness to the patient. The great
point, however, in this practice, is the
continuation of the dressings, without
which, all our efforts will prove inefficient.
They should never be removed until it is
absolutely necessary, either from the ex-
cessive discharge from the wound soiling
the cotton, or the insupportable fetor
which may arise from it: this last in-
convenience, however, may be corrected
by the chloride of lime. Many surgeons
have attempted this practice, but, igno-
rant of the necessity of continuing the
dressings for several days, have been
quite unsuccessful. The reason of this
is obvious:?The cotton adheres with such
tenacity to the raw surface of the wound,
that, in removing the dressings daily, it
cannot be accomplished without produc-
ing a considerable degree of irritation, and
disturbance of the restorative pi'ocess. It
is of importance to observe that, when a
part of the dressings are soiled, it can be
taken away without removing the whole
of the dressings. In the cases which have
been treated in this infirmary, the dres-
sings have been removed about once in
six or eight days on an average. Every
one of the above-mentioned individuals
experienced immediate relief from the
application of the cotton, and progres-
sively recovered, without exhibiting the
slightest constitutional irritation.
I have no doubt that this treatment
prevents, in a great measure, those de-
terminations to the viscera, which are so
frequently consequent on extensive burns.
Cotton has not been applied exclusively to
burns, it has been rendered subservient
to the cure of abrasions, and superficial
injuries of every description, and will, no
doubt, ultimately become a general ap-
plication.
1828] Mr. Brodie's Case of Popliteal Aneurism. 541
0. ST. GEORGE'S HOSPITAL.
Mk. Brodie's Case of Popliteal
Aneurism.
In the month of August, the Lancet gave
' an account" of a case of popliteal
Aneurism at that time under Mr. Brodie's
care in St. George's Hospital, in which
" account," as usual, there were errors
m abundance. In No. 7 of the Qazette
the authentic version of the case was
published, and the blunders and mis-
statements of the Lancet exposed. In
our second fasciculus we gave an abstract
of the case, and copied almost verbatim
from the Gazette the contradictions of the
Said misstatements. To this expose, the
"Invaluable" of last week has made
a reply, and that in such a ludicrously
intemperate style, that it irresistibly
reminds one of a puddle in a storm.
It is a circumstance of which our pro-
fession should be justly proud that in the
improved mode of conducting medical
controversies in the present day, we are
not trammelled by the obsolete and vul-
gar rules of truth and justice. Thus, if a
man convicts you, in plain English, of a
falsehood, all you have to do is to call
him a bat, or a cock-sparrow, and that
settles the matter. In the present in-
stance, then, the Lancet does not even
attempt to defend the truth of its allega-
tions because it knows, full well, they
are indefensible. No, this impartial
journal being convicted of mis-statements
turns round, and like the ragamuffin in
the streets, pelts Mr. Brodie with hand-
fuls of abuse !!
First, it assumes that which it has no
right to assume, and which assumption
we know to be untrue, viz : that Mr.
Brodie is the writer of the Report, merely
for the purpose of visiting upon him the
sins, such as they are, of the Reporter! It
then gives a series of themost quibbling and
contemptible criticisms upon the wording
of the case which we ever remember to
have seen even in the pages of the Lan-
cet. We would not insult the readers
of this journal by going seriatim into
these paltry verbal criticisms, but we
will notice one or two of them. The
Lancet is absolutely amazed that the
aneurism should increase " by the patient
being greatly harrassed in mind and
body," and calls it a singular position I
Why this learned Theban had better get
him to his book as soon as possible, if
he is so ignorant as not to know that exer-
tion will aggravate an aneurism, and thut
rest is indispensible to its cure. The
Lancet takes mortal offence at the words
"seem," "numb," and " analogous*"
and doubtless its criticisms are of the
highest importance in a pathological
point of view, and surprisingly just if one
could but understand them?as, however,
this is a hopeless matter we shall pass
over these abstruse questions and come
at once to the " practical points."
On the 6th Sept., fourteen days after
the separation of the ligature from the
femoral artery, a haemorrhage of fluid
blood burst in a stream from the wound.
Firm pressure was steadily made upon
the groin, but in spite of it there was a
" threatening of bleeding in the night,"
and, on the morning of the 8th, the pres-
sure being still continued, haemorrhage
took place " to a greater extent." The
tourniquet was then applied, but, after a
time, on loosening it a little, an oozing was
observed, and Mr. Brodie (very properly
in our opinion) " thought it advisable to
tie the artery in the groin," which he did
at 2, p. m.
Now, after this statement, which the
Lancet has transferred to its own pages,
it absolutely makes the following asser-
tions. " The loss of blood here had been
trifling, and the obvious measure of com-
pression does not appear to have been
tried. What, was the two days pressure
on the groin nothing ? was the applica-
tion of the tourniquet nothing ? was the
constant dread, and repeated occurrence
of the bleeding from the morning of the
(ith till 2, p. m., of the 8th nothing ?
' As Mr. Abernethy says, there is such a
thing as common sense. The Lancet
makes a sad outcry at Mr. Brodie for hav-
ing tied the artery, but let us see what it
has itself proposed. " A graduated com-
press should have been placed over the
wound, and the trunk of the artery, from
the groin to the wound, should have been
covered by another. These should have
been covered by a bandage from the toes
to the groin." ! ! ! To say nothing of the
awful size of these compresses, we shall
just look a little at the state of the limb
and the wound, which were to have been
tortured by all this bandaging. The re-
port states that spongy and indolent
granulations had arisen from the wound,
542 ? Periscope; or, Circumspective Review. [March.8
which was little disposed to heal, and
that the thigh around was so swollen as
to require the application of a poultice.
In such a condition a bandage from "the
toes to the groin " with compresses, " to
match" would be admirable, most ad-
mirable, practice truly.
There is one other point on which we
shall merely touch, as the consideration
of it would require more space than we
can at present afford, namely, the ap-
plication of the second ligature just be-
low the giving off of the profunda. It
is argued by many, and we believe the
argument a sound one, that the tying of
a main trunk near the origin of a very
considerable branch, is unsafe, inasmuch
as the circulation is brought up to within
a short distance of the ligature?there is
no sufficient clot in consequence, and
the danger of secondary haemorrhage is
thereby increased. We say that we be-
lieve the argument a sound one, but yet
it is disputed, and the fact very far from
being unequivocally established. This
being the case, we should like to know
what right the Lancet has to abuse Mr.
Brodie for doing that, which Sir A.
Cooper, Baron Dupuytren, and, we be-
lieve, Mr. Cline, have done before him,
and which, for ought we know, surgeons
may be doing daily. The Lancet arrays
against Mr. Brodie, an observation of
Mr. Hodgson's, but it has forgotten in its
hurry, to notice another fact by the same
gentleman, as militating against the ne-
cessity for a coagulum I
MISCELLANY.
1. Carbonate of Potash and Opium,
in Traumatic Tetanus.
Many of our readers will remember that,
a few weeks ago, Dr. Barry mentioned,
in the Westminster Medical Society, a
practice which he had tried, with success,
in traumatic tetanus, while serving with
the Portuguese troops in the Peninsular
war. This practice was, alternate doses
of carbonate of soda and opium. The
alternation-of these medicines was sug-
gested to him by another gentleman,
who probably learnt it in Germany, where
it has got the appellation of the " method
of Stultz." A case has lately been pub-
lished in Hufeland's Journal, and copied
into the Revue Medicale, which eluci-
dates this practice.
Case. A young woman of 19 years of
age, robust and healthy, received a con-
tused wound on the instep of the left
foot?for which nothing was done by any
surgeon. A month after the accident,
and before the wound was healed, she
was suddenly seized with trismus, which
went on to opisthotonos. The physician
who was called in, found the patient in
this state, the wound in the foot being in
a bad condition, and covered with proud
flesh. Thirty-two ounces of blood were
abstracted?purgatives were given?and
poultices were applied to the wound.
The bowels being opened, the method of
Stultz was put in force, and, in the course
of twelve days, she took 224 grains of
opium, in alternate doses with carbonate
of potash, the quantity of the latter not
being mentioned. Under the influence
of this treatment, the spasms became
less severe and less frequent, till a general
swelling of the body came on, when
they ceased altogether. This swelling
appeared to be of an anasarcous nature,
and gave vvay to diuretics. It was suc-
ceeded by an eruption on the skin re-
sembling scarlatina. This last disap-
peared, and the patient got quite well.
The rationale of this practice, we be-
lieve, has been attempted on the suppo-
sition that, the alkali enables the opium
to get at the nervous surface of the sto-
mach much more readily than it otherwise
would do?and that, in shqrt, it is the
preparation which gives the latter remedy
the complete power of acting. As opium
is still the medicine on which reliance is
chiefly placed in this terrible disease, and
as there appears no counter-indication to
the alkali, we think the method should
be tried.
2. Pathological Investigations in
the Dissecting-room.
Not unfrequently bodies are brought into
the dissecting-room presenting patholo-
gical appearances worthy to be recorded,
although, unfortunately, we thus become
acquainted only with the phenomena in
the dead body, not with the symptoms
which indicate them < in the living. In
182S] Pathological Investigations in the DissectingJjIoom. 543
the Bibliotheque for November last, M.
Cruveilhier has published an account of
a dissection, which we shall notice here.
Varices of the Veins of the Round
Ligament.
Tt is well known that the veins of the
spermatic cord in the male, are subject
to a varicose enlargement, and that this
affection, has been occasionally mistaken
for an inguinal hernia. We remember,
a month or two ago, seeing a case at one
of our hospitals, where a young man in a
drunken fray got a blow on, or sprained
his right groin, in which a tumour im-
mediately appeared. Some few hours
afterwards he was admitted into the
hospital, havingall the symptoms of stran-
gulated inguinal hernia, which was treated
by the warm bath, bleeding, the taxis, &c.
without x-elief. The operation was tixed
for 4 p.m. but in the mean time, cold,
by means of the sulphuric aether, was
steadily applied. The surgeons and pu-
pils had collected, and all was prepared
for the operation, when a portion of gut
suddenly " went up" under a moderate
application of the taxis. Considerable
swelling, however, still remained, and it
eventually turned out, that there had been
an inguinal hernia, combined with vari-
cocele and hernia humoralis, the patient
having laboured under a clap for some
time previously.
The patient, we should say the subject,
which came into the hands of M. Cruveil-
hier, was a female, about 60 years of age,
having an oblong tumour at each ex-
ternal ring, which M. C. imagined to be a
brace of hernias, and proceeded to operate
on one of them accordingly. On cutting
through the skin, the external pudic
veins were found to be very tortuous,
and one large vein in particular was seen
to cover the upper pillar of the ring.
Continuing the incision, the knife came
down to a muscular substance, resembling
a hypertrophied bladder, or uterus at the
3d month of pregnancy. M. Cruveilhier
thought he had come upon a hernia of
the bladder, but n'importe he cut on,
divided the " fleshy tissue" into laminae,
and at last opened into a large vessel,
which proved to be a vein with its coats
much thickened. The operator was now
fairly bothered, and determined to satisfy
his doubts by cutting into the abdomen,
and introducing his linger from that into
the ring. To shorten a long story, it was
discovered that the tumour was made up
of the " muscular substance" of the
round ligament considerably thickened
and hypertrophied, and its veins bound
together by fibrous bands, and exceed-
ingly varicose. The veins of the legs
were very varicose also.
This good lady must have been a mu-
seum in herself, for besides the above
rare affection of the round ligament, she
presented some curious appearances ia
the abdomen. The mesentery commenced
at the pylorus, so that the duodenum was
contained within its lamina:, and not
distinguishable in this respect from the
small intestine generally. The right half
of the pancreas was contained in the
mesentery, and the caecum, ascending,
transverse, and descending arch, and
sigmoid flexure of the colon, formed but
one tortuous arch, not above half its
usual length. Several other anomalies
in the mesentery and epiploon, were
observed, but we need not stop to des-
cribe them. The lower half of the an-
terior Avail of the vagina was affected
with cancerous ulcerations, by which it
communicated with the bladder. The
base of the ulceration was white, brittle,
and prominent, several little prominences
projecting into the bladder, and one of
them being covered by a calculous deposit
of uric acid.
Whilst upon the subject of dissecting-
room pathology, we may take notice of a
paper published by Mr. Csesar Hawkins,
in No. 10 of the Medical Gazette, con-
taining an account of some appearances
observed in two bodies brought into the
School of Great Windmill-street. The
first was a middle-aged man, who had
been affected with ricketts to an extreme
degree. It appeared that, whilst labour-
ing under this disease, the left knee had
been dislocated, so that the tibia rested on
the fore-part of the femur. In this situa-
tion, a new joint had been formed, the
back part of the head of the tibia being
flattened, and a kind of cup having
formed on the front of the femur, above
the condyles, which was rendered more
complete by the growth of a large knob
of bone immediately above it. The sur-
faces of this new articulation were coated
with an imperfect cartilage, and pro-
vided with a distinct synovial membrane,
and ligaments of considerable strength.
514 "?* Periscope ; or, Circumspective Review. [March 8
The patella of that side was much smaller
than the other, probably, as Mr. Hawkins
observes, from its not having been called
into exercise, the new form of joint not
allowing of flexion and extension of the
leg. The hip-joints were remarkably
altered also, the neck of the femur being
on each side absorbed, so that the tro-
chanter and head of the bone were on a
level with each other. The head itself
was flattened and widened, as was the
acetabulum, which was much shallower
than usual. From these circumstances,
vMr. Hawkins thinks it is evident that the
enarthrotic, or ball and socket articu-
lation was lost, and had degenerated into
little more than a mere hinge-joint.
The patient had been obliged to use
crutches, so that the head of the humerus
was forced up against the acromion, and
between these parts there was found a
number of enlarged bursse, filled with a
quantity of thick gelatinous fluid. Thus,
the very pressure and irritation produced
their remedy, namely, the interposition of
these additional friction-wheels between
the ends of bone.
In the second case aetailed by Mr. H.
it appears that the median nerve, flexor
digitorum sublimis, and flexor carpi ra-
dialis muscles, and the radial artery, had
been divided by some wound inflicted a
considerable time previous to the pati-
ent's death. The portions of the flexor
digitorum were united by an imperfect
ligamentous substance, and the flexor
carpi was partly deficient. The radial
artery was likewise deficient for nearly
three inches, its lower part being sup-
plied by an enlarged branch of the in-
terosseous. But the more interesting
appearances were observed in the median
nerve. This terminated, about the mid-
dle of the fore-arm, in an oblong tumour
of a light brown colour and firm consist-
ence. The filaments of the nerve were
separated, and spread out over its up-
per end, whilst at its lower, the tumour
had contracted inseparable adhesions to
the ligamentous part of the flexor subli-
mis. The nerve below the annular li-
gament was perfectly natural, but be-
tween this lower portion and the tumour
on the upper, there was a separation of
at least three inches, without any inter-
vening substance whatever. An enlarged
branch, however, was given off from the
?superficial division of the muscular spi-
ral, which passed through the flexor
sublimis and joined the lower portion of
the median nerve. One or two smaller
anastomoses were also observable be-
tween these nerves nearer the wrist.
3. Extract of Valerian, in full jdoses.
Every practitioner is aware that valerian
exerts a very considerable sedative influ-
ence over the nervous system ; but the
extremely disagreeable odour of that root
(to some more offensive than assafoetida)
very much limits its use, especially among
females, where it is most wanted. Dr.
Guibert, of Paris, has recently exhibited
the extract of valerian, in doses vai-ying
from one to two or three drachms pel'
diem, in several disorders of the nervous
system?and, if the cases detailed be
faithful, with considerable success. As
the medicine is perfectly safe?as the
root has long been employed with ad-
vantage?as its bulk and flavour are ob-
jectionable?and as the extract appears
to possess all the medicinal properties of
the root, divested of several inconveni-
ences, we think it is a medicine which
deserves attention, particularly in a num-
ber of female complaints equally distress-
ing and unmanageable.
The cases in which Dr. Guibert has
employed the extract are spasmodic con-
tractions of the muscles of the limbs??
nervous tremors in adults, when unac-
companied by any signs of plethora??
chorea?epilepsy, in which disease he
has found the valerian very serviceable,
after sanguineous and other evacuations
?palpitation of the heart, when of a
nervous character, as they very often are
?spasmodic asthma, and nervous dyspnoea
?hooping cough, in its later stages, and
after the inflammatory symptoms have
subsided?dyspepsia and gastrodynia un-
attended with any obvious inflammatory
symptoms?vomitings of a nervous cha-
racter, and not apparently dependent on
any organic disease?and last, not least,
the various proteian forms of hysteria. Of
all these complaints, the Doctor has ad-
duced examples in our Parisian cotem-
porary?the Revuf. Mkdicale, for De-
cember last. These cases we shall not
detail; but we think the remedy is worthy
of the English practitioner's notice.
1828] Diabetes Mellitus. i. 545
4. Diabetes Mellitus.
A late meeting of the Westminster Me-
dical Society was occupied with the sub-
ject of uiabetes. Dr. Ayre (to use the
learned language of the Lancet) "pre-
faced the relation of a case with a Pero-
ration," which we would call an exor-
dium, on the pathology of the disease.
The Doctor did not agree with Heberden,
that it was a breaking up of the constitu-
tion in advanced life?nor with Cullen,
that it was nearly always fatal?nor with
those who considered it to be dependent
on a faulty digestion,?an imperfect as-
similation,?or a bad sanguification. In
short, he looked upon diabetes to be a
local disease or disorder of the kidneys?
the nature of which, he thought, was
chronic inflammation. Entertaining this
view, he therefore expected but little
from any peculiar diet tending to prevent
the formation of saccharine matter in the
fluids, and its elimination by the kid-
neys. Local detractions of blood from
the loins formed almost the whole of his
therapeutics. He stated the details of a
case, part of which had been drawn up
by the patient himself, from which it
appeared that the quantity of diabetic
urine amounted, at one time, to more
than 20 pints per diem, while the thirst
was unquenchable, and the appetite vo-
racious. The plan of treatment above-
mentioned was put in force, and by the
time the patient had been seven times
cupped on the loins, the disease was sub-
dued (or supposed to be so) and the
health restored. Unfortunately for the
efficacy of this plan, Dr. Barry informed
the Society that the same patient had
come under his care, shortly after he left
Dr. Ayre, and the diabetes had returned
nearly as bad as before. Dr. Barry had
put the gentleman on Rollo's plan of
animal food diet, and to this the disease
was once more giving way.* A sharp
discussion now took place respecting the
chemical pathology of diabetes?Dr.
Barry maintaining that the grand source
of the malady would be found in the
fluids, while Dr. Ayre, Mr. North, and
some others, considered the solids in
fault. Mr. North brought forward the
authority of Berzelius to prove that, in
certain affections of the liver, the urea
disappeai's from the urine, and a saccha-
rine principle is there evolved?-in short,
diabetes produced.
Dr. Johnson was inclined to agree with
Dr. Barry, that the pathology of diabetes
could not be affixed on any one particu-
lar organ?neither the kidneys, the liver,
the stomach, or the lungs, exclusively.
In all the severe cases which he had
seen, however, there was some evident
functional disorder of the system accom-
panying the diabetes. In every case
where he had an opportunity of examin-
ing or seeing examined, the body after
death, the lungs were found affected with
disease, whatever were the appearances
in other organs. In some researches
which he made among the most authen-
tic dissections that have been recorded
of this disease, he found that, in very
few instances indeed, were the lungs in
a state of integrity. By this he did not
mean to infer that disease of the lungs
was the cause of diabetes; but he
thought that this fact authorised us to
suspect a considerable connexion be-
tween disordered function in the respi-
ratory apparatus, and the production of
diabetic urine. In respect to treatment,
he had found restriction to animal diet,
with occasional blood-letting, and great
attention to the improvement of such
functions as appeared to be deranged,
the most successful plan. The general
sense of the Society appeared to be in
favour of the mode of treatment recom-
mended by Rollo. Dr. Ayre adhered to
his opinion, that the depletive plan of
Watt, was superior?and especially local
depletion, from the neighbourhood of
the kidneys. It appeared, indeed, that,
in the case detailed by Dr. Ayre, even
admitting that the result was not con-
clusive, the cupping on the loins always
gave temporary relief. This the patient
himself acknowledged to Dr. Barry,
though he got tired, he said, of the plan,
as the relief was transient.
5. Surgical Curriculum.
In our last Fasciculus we intimated our
intention of examining seriatim, the dif-
ferent items in.the recent curriculum of
* This discrepancy was afterwards satis-
factorily explained.?Ed.
Vol, VIII. No. 16. 3 N
546 Periscope;, or, Circumspective Review. [March S
the College of Surgeons. Entertaining,
as we do, the greatest respect, and even
friendship, for several individual mem-
bers of the Council, and knowing that
many of them are anxious to do every-
thing that is liberal and conducive to the
public good, we cannot but hope that the
observations of those who must be dis-
interested on the subject will be taken in
good part, and not attributed to sinister
motives, or a disposition to hypercritical
censure. In our remarks, we shall ap-
peal entirely to the judgment, and not to
the prejudices, of all concerned. In the
present instance, we shall limit our in-
quiry to the first clause of the curriculum.
1. "The only Schools of Anatomy
and Physiology recognized, are,
London, Dublin, Edinburgh, Glas-
gow, and Aberdeen."
We should have thought that the liberal
opinions lately introduced by many?in-
deed we might say by all, the most
talented and enlightened men of the age,
in respect to free trade, and international
reciprocities of interest, would have ope-
rated on the minds of men of science
and literature, and that they would not
have enlisted themselves on the side of
ignorance, narrow-minded policy, John-
Bull prejudice, and downright opposition
to the advancement of that science and
literature, of which they are, at once, the
guardians and the ornaments. And, of
all branches of human knowledge, that
of medicine and surgery should surely
be the most entirely emancipated from
local, or even national restrictions, seeing
that it is the same in kind, on every point
of this great globe which we inhabit.
We have no objection to that patriotism
which aims at raising the character and
acquirements of one nation over those of
other nations. This is laudable, and tends
to useful emulation among all. But we
most decidedly object to that principle
Ayhich limits or impedes the means of
acquiring that, knowledge which, raises
the character of individuals, and con-
sequently of nations. What would be
thought of the Russian Autocrat, if he
threw every kind of impediment in the
way of his naval, military, and medical
subjects, .who sought to improve them-
selves in ship-building, military disci-
pline, and the practice of physic aud sur-
gery, by sojourning in Paris, London, or
Vienna ? We would call him a Goth, a
Tartar, or a Vandal! But he has not
permitted us to accuse him of such bar-
barism. He has encouraged every indi-
vidual exertion of this kind?not only by
withdrawing every obstruction, but by
holding out positive rewards. Yet we
consider all medical knowledge as limited
to the soil of Britain?and instead of fos-
tering the praiseworthy endeavours of
individuals to draw professional lore from
every spot where it can be procured, we
issue our little injunctions against all
science that is not taught on half a dozen
points or dots on the surface of the Bri-
tish Isles ! How is this reconcileable with
sound sense ? How does it quadrate with
a maxim as old or older than the Chris-
tian sera?" fas est et ab hoste doceri."
When we reflect on the liberality, the
enlightened policy of the great medical
schools of Europe, where English study
is allowed to form part of the curricu-
lum, we blush for the institutions of our
.own country, which enact statutes that
would do no credit to the intellects of a
Cossack, an Otaheitian, or Canadian!
We know, indeed, that there are men,
connected with our chartered bodies, who
have the effrontery to stand up, even in
our medical societies, and whine forth,
with methodistical cadences, their little
interested orations about the excellence
of medical jurisprudence in this country,
and the complete superiority of British
pathology over that of every other people
in Europe. They are aware that this
flattering unction will gain the smile of
the moment, from those who are engaged
in the same line of policy as themselves ;
but, as long as our hand can wield the
pen, we will bring this " mentis gratis-
simus error," before the bar of public
reason, for adjudication.
What is the collegiate excuse for this
restriction of anatomical and pathological
study to certain schools in this country ?
The old corn-law policy. Favour the
English farmer. Protect the farmers of
anatomy and surgery in Guy's and Bar-
tholomew's, although the consumers, the
students, may pay twelve or fifteen guineas
for each body, and consequently may be
half-starved during each season ! But why
do the anatomical farmers import their
wine from the Bordelais, when grapes
are seen mantling on the walls of their
1S2S] Inguinal Aneurism. ' 547
country-houses ? Because the vine grows
plentifully in the fields there, and can
only be trained on the sunny sides of their
houses here. It is the same with ana-
tomy. The dear and scanty supply in
our dissecting rooms must be put up with,
and preferred to, the cheap and plentiful
supply in Paris?and all this for the en-
couragement of the British anatomical
farmer ! The illiberality of this policy is
undeniable, and what are the advantages,
after all, which the monopolists can hope
to gain by it ? There is not one in one
hundred of English students who has
the means of travelling to other countries
for anatomical, pathological, or clinical
improvement. Yet to deter this one in
two or three hundred, from prosecuting
a portion of his studies in those places
which are peculiarly favourable, the coun-
cil of the College has enacted a law which
must be viewed by every enlightened man
in Europe as a libel on the English pro-
fession ! The sting of this libel, too, is
rendered the more galling, when we see
such a place as Aberdeen "recognized,"
while Paris is " incognizable !" Was it
possible that the members of the Council
could preserve their gravity when they
put their seal to this primary and funda-
mental law ? What reason has been as-
signed for giving the prerogative to Aber-
deen, in preference to twenty other and
better places ? Because, forsooth, there
is an anatomical chair in Aberdeen!
Why so there is in Oxford and Cam-
bridge, and yet we do not see these
" venerable seats of learning," recog-
nized by the College. Now anatomy and
pathology may be taught by a chair or
by any other wooden machine?but it
can only be learnt by dissection. Dissec-
tion cannot be performed without bodies
?and bodies cannot be procured, except
where there is an abundant population
and consequent mortality. If these be
axioms, and we believe they are, the
statute which recognizes Aberdeen, as a
school of anatomy and physiology, and
rejects Manchester, Liverpool, and other
great provincial cities and towns, where
hospitals abound, bodies can be procured,
and excellent anatomists reside?is a blot
on the justice and common sense of the
country. The sooner the College revise
and amend this first item in their curri-
culum, the better. It would be only de-
cency as well as liberality to admit one
year, at least, of Parisian or other conti-
nental time in the curriculum?it would
be good policy, inasmuch as it would hold
out some little encouragement for young
men to extend their knowledge and expand
their minds, by visiting the institutions of
foreign countries, where clinical practice
is open to strangers, and anatomy can be
prosecuted at a very trifling expense, in-
stead of binding them down to study ana-
tomy, where dissection is either imprac-
ticable for want of subjects, or ruinous by
the expense of them. So much for the
first count in the indictment. We shall
discuss the other counts in succeeding
fasciculi of this Journal.
6. Inguinal Aneurism?External Iliac
Artery tied. By Mr. Brodie.
It is well known how very successful the
operation of tying the external iliac has
proved in the hands of surgeons, both in
this country and abroad. It is to Mr.
Abernethy that we are indebted for it,
and really the history of the successive
attempts made by that gentlemen reflects
the highest credit upon his firmness and
abilities. At the time Mr. Hodgson's
valuable Treatise on Aneurism was first
published, namely, in 1815, twenty-two
cases had occurred, of which fifteen had
proved successful. Since that period the
operation has been frequently repeated,
and, upon the whole, with great success,
so much indeed, that it has been pro-
posed to have recourse to it in popliteal
aneurism, a plan which is not very likely
to be put in practice, or, if put in prac-
tice, to be of service.
The subject of the present case, which
we had an opportunity of witnessing, and
shall detail very briefly, was a tailor,
set. 38, who had been exposed to damp
and cold in White-Cross Prison, and had
suffered a great deal of anxiety on ac-
count of his family. In November last,
Avhilst having connexion with his wife,
he felt as if something had given way in
the left groin, and soon afterwards no-
ticed a small throbbing tumour in the part.
It increased gradually for a fortnight, then
remained stationary until the latter end
of January, when it again began to swell,
and the limb became cedematous. When
admitted into St. George's, February
548 Periscope ; or, Circumspective Review. [March 8
15th, the aneurismal tumour was large,
apparently extending the whole length
of the common femoral artery; rather
triangular in form, occupying the trian-
gular space between the sartorius and
pectinseus ; reducible, but not removeable
by pressure ; bounded abruptly above by
Poupart's ligament; and pulsating very
forcibly. Pressure upon the external iliac
arrested the pulsation, and diminished
the tumour, whilst pressure on the super-
ficial femoral below* lessened the pulsa-
tion, but had little or no effect upon the
tumour. The whole limb was swollen
and tense, the foot (edematous and numb.
There was much pain in the direction of
the external branches of the crural nerve;
the countenance was sallow and anxious;
the appetite indifferent ; tongue red;
bowels costive ; pulse quick and hard.
Under these circumstances, he was placed
in bed, bled once or twice, and purged,
until the 21st, when, being in a more
favourable state for the operation, and
rather anxious for its performance, the
external iliac was tied by Mr. Urodie.
We were present at the operation, and
must say that it was performed with a
great deal of coolness and facility. The
plan adopted, was that of Mr. Abernethy's,
with some little modifications, and the ar-
tery was readily discovered and secured,
but, in consequence of the vessel having
contracted some adhesions to the sur-
rounding parts, care was required in
passing the ligature around it. A thin silk
thread was employed, one end cut rather
short, and the other brought out at the
external wound, which was brought toge-
ther by sutures and adhesive straps. Jn
the evening, the pulse had got up a little,
and at 12 p.m. next day, he was labouring
under the following symptoms. Counte-
nance anxious?breathing hurried and
difficult?pain in the right side, increased
on making a deep inspiration, speaking
loud, or coughing?pulse corded and
full?surface covered with perspiration?
tongue white and thickly coated?thirst
?confined bowels. He was bled to
oz. xviij. with the effect of relieving the
pain and diminishing the anxiety of coun-
tenance. He was likewise directed to
to take a senna draught every six hours
until it operated. From this time no un-i
favourable symptoms had occurred up to
the 28th, when we saw the patient. He
still laboured under some cough, but it
was unattended with pain in. the side, or
hardness of pulse. The countenance had
lost the anxiety which marked it prior to
the performance of the operation?^the
pain in the thigh had entirely disappeared
?the swelling of the limb was subsiding,
and the wound was going on very favour-i
ably. It may be mentioned, that on
drawing the ligature, the pulsation in
the tumour ceased, and never afterwards
returned, but the tumour did not very
materially diminish in size at the time.
Since that, however, it has gone on
steadily decreasing and a pulsation is
distinguishable in the superficial femoral,
as well as in another branch on the inside
of the knee, probably the anastomotica
magna.
It will be observed that Mr. Brodie
adopted Mr. Abernethy's mode of ope-
rating, in preference to Sir Astley
Cooper's. In a clinical lecture on the
case, Mr. B. assigned as a reason, that
he had twice done the former with suc-
cess, and therefore was more au fait at it,
and that as he had reason to believe the
lower portion of the external iliac impli-
cated in the disease, he naturally adopted
that mode of operating, which would
enable him to arrive at the upper portion
of the artery. There is another disad-
vantage attending Sir A. Cooper's method
(viz. a semi-circular incision carried al-
most transversely beneath the internal
ring) which is this, that there is a danger
of wounding the epigastric artery. This
accident happened to no less a personage
than the French " Leviathan of Surgery"
M. Dupuytren, in 1821, and the patient
died of peritoneal inflammation. These
were some of the reasons assigned by
Mr. B. for operating as he did, but
there was yet one more, and that a
forcible one. In the low, or Sir A.
Cooper's, operation, the ligature is ap-
plied just above the giving off of the
epigastric and circuinflexa ilii, and the
conscquence is, that the plug may be so .
* Mr. Hodgson, in his Treatise, asserts
that pressure on the artery below renders
the tumour more tense, and the pulsation
more violent ; indeed, he recommends this
as a diagnostic of aneurism. From this re-
port, for the correctness of which we
pledge ourselves, it is evident that Mr.
Hodgson made too sweeping a statement.
1S2S] Wound of the Knee. 549
inconsiderable between, the ligature and
these vessels, as to afford no sufficient
security against secondary haemorrhage.
This is no visionary objection, for in a case
in which M. Dupuytren tied the vessel at
this situation, in the Hotel Dieu, the pa-
tient nearly sank under secondary haemor-
rhage evidentlyproeeeding from the lower
portion of the artery. On examination,
M. D. perceived the epigastric to have
become enormously enlarged, and to
have brought the blood round almost to
the verge of the ligature, on the sepa-
ration of which, the adhesion between
the inner coats of the vessel gave way,
and bleeding was the result. In another
instance, Sir A. Cooper tied the femoral
artery, immediately below the origin of
these branches, the epigastric and circum-
flexa ilii, and in that case also secondary
haemorrhage followed. The same thing,
we believe, happened to Mr. Cline y and
in the case at St. George's Hospital,
upon which so much valuable indignation
has been lately thrown away, the ligature
is said to have been applied a little below
the giving off of the profunda, and it is
not unreasonable to conclude that this
circumstance favoured, if it did not ac-
tually occasion, the occurrence of the
secondary haemorrhage.
In the report it will be observed, that
the subsidence of the tumour did not im-
mediately follow the application of the
ligature, and this, we believe, is always
the case in what Scarpa calls " false
aneurism." In the " true aneurism,"
which is merely a dilatation of the vessel,
and which if let alone, will very often
remaih stationary for a considerable length
of time, or even during the patient's life,
if it be an old person, the contrary is the
case, the tumour generally collapsing re-
markably after tying the vessel. This,
we apprehend, will afford a key to several
circumstances connected with some of
those operations on the arteries, which
have lately astonished the surgical world-.
The bleeding practised in this case on
the day after the operation, was bold and
decisive, and was, in all probability, the
means of saving the patient's life. The
pleuritic affection with which he was
attacked w?s insidious, but not the less
dangerous, and had it not been " put out"
at once, as it was by the decisive blood-
letting, it might soon have proved more
than a match for the lancet, or any other
remedy. These inflammations of the
pleura, all know, are not uncommon
after severe injuries or operations, and in
the case of ligature of the carotid, re-
corded in our third fasciculus, it carried
oft' the patient in a very days.
We see nothing in the details of the
case which would lead to an unfavourable
prognosis. The ligature is yet to come
away, but there is no good reason, which
we are aware of, to apprehend secondary
haemorrage on its separation. However,
as the French say?nous verrons.
7. Wound of the Knee?Extensive
Suppuration?Amputation op the
Limb.*
This case occured under Mr. Travers,
and, as far as we can understand it,
(which, from the manner in which it is
drawn up, is no very easy matter) the
particulars are as follow.
The patient, set. 52, of " lax fibre "
and intemperate habits, received a lace-
rated wound over the patella, denuding
the bone, but not opening into the knee-
joint. This was on the 23rd, and on
the 26th Dec. the wound was granu-
lating, and all was well, save that at the
lower part of the patella, there was " a
dark coloured slough, the size of a shil-
ling, partially adherent." He was or-
dered quinine, porter, and diluted nitric
acid lotion to the part, and the next date
of report is Jan. 30th, upwards of a
month. At this time the slough had
partially separated, so that it must have
been almost in statu quo ; the bone was
exposed; there was severe pain in the
joint, and on pressing the side of the
wound, fetid pus escaped. Pulse small
and quick?bowels costive. Wine and
opium were administered, but an open-
ing formed on the outer side of the joint,
leading into its cavity, and discharging
a quantity of dark-coloured, offensive
pus, and on the 5th Feb. the leg and
foot were osdematous?the integuments
inflamed and painful?discharge from
the joint profuse?and the pulse small,
weak, and innumerably rapid. These
symptoms are noted down with the most
careful ambiguity, but still they seem to
* Lancet, No. 234
550 Periscope; ok, Circumspective Review. [March 8
furnish an idea, faint and glimmering
we own, of the characters of an affec-
tion, termed by nomologists erysipelas;
at any rate, if the osderna, and inflamma-
tion of the integuments did not depend
on erysipelas, we confess that we are
totally unable to divine what they did de-
pend upon. Under these circumstances,
amputation of the thigh was proposed,
and performed by Mr. Travers, at its
middle, in consequence of the " cuta-
neous inflammation " having extended
to its lower part. On examining the
amputated limb, the leg and foot were
found enormously enlarged,?a large
gangrenous patch, surrounded by a livid
redness, occupied the calf, and there
were several vesicles at the upper part
of the leg. On making an incision into
the limb, a mixture of pus and serum,
escaped from the subcutaneous cellular
tissue, as well as from that connecting
the various muscles. The joint contained
much offensive pus?its synovial mem-
brane was highly inflamed?its cartilages
softened, and in one part ulcerated, and
the patella was softened, and " partially
detached." The patient, on the second
day after the operation, became delirious
?there was pain in, and profuse dis-
charge from the stump, which was of a
dirty ash-colour, and, on the morning of
the 11th, he sank.
This report is so confused, and the
language so unlike what is commonly
known as the King's English, that we
cannot be at all sure of the exact nature
of the case ;?as far, however, as we can
judge, it appears to have been an attack
of " gangrenous erysipelas " occurring,
as that terrible disease generally does
occur, in a person of weakened frame,
and intemperate habits. Whether the
suppuration within the joint, preceded,
accompanied, or succeeded the erysipe-
latous inflammation, is not particularly
clear, but, whichever was the case, it
is obvious that amputation was, indeed,
a dernier resort, and, moreover, a very
hopeless one.
8. Curious Specimen of Ratiocination.
The Profession is well aware that the
Lancet has plumed itself for the illumi-
nation which it has been able to throw on
the non-professional public, anil on the
jury in Mr. Stanley's case, by which the
said non-professional public are taught to
place no reliance whatever on the profes-
sional skill or veracity of a Cooper, a
Brodie, a Bell, a Travers, an Abernethy,
&c. By proving that such men are igno-
rant, the Lancet sagaciously concludes
that the public will, at once, decide that
surgeons in general are infallible?that,
in fact, the detection of an error in the
former class will for ever absolve the
latter from all suspicionof such weakness!
" As to the effect (says the Lancet)
produced by this trial on the minds of
the public, it is decidedly favourable to
the great body of the Profession. It is
calculated to raise the great mass of
English surgeons, absurdly denominated
general pkactitioners, in public esti-
mation."*
This specimen of reasoning has no
parallel in the annals of medical litera-
ture ! Those men who have arrived at
universal eminence in their profession?
by whom a great portion of the Pro-
fession has been taught?whose opinions
are sought after and respected, both by
medical men and by the public at large?
these men, we say, are to be held up to
ridicule, as totally ignorant?and, as a
natural corollary, all those who have de-
rived their opinions and practical precepts
from these men, must rise in public
estimation ! One'would really suppose
that the Lancet considers its readers as
totally incapable of a single exertion of
* We would ask the Lancet where is
the absurdity, or the degradation of the
term "General Practitioner?" It
is that under which Hippocrates, and
some of the most eminent in our pro-
fession have practised. It supposes a
general acquaintance with all branches
of the Profession?and without which
acquaintance, no surgeon, no physician
can be a good practitioner. This sentence
of the Lancet is the greatest libel ever
penned against the " General Prac-
titioner." The Lancet is for ever run-
ning out against " pure surgeons," and
now it turns round and ridicules the term
" General Practitioner," by which it
must advise the change to " pure sur-
gery such as it is practised by Thomas
Wakley, William Lawrence, and James
Wardrop.
1828] Mr. Stanley's Trial. 551
thought?as completely devoid of every
particle of intellect! The conclusion
which the public must draw from all
this (as far as the public can be influ-
enced by such a tool as the Lancet) is
this?that we are, one and all, from the
highest to the lowest in the Profession,
a set of block-heads?ignorant of our
Hit?and leagued to screen each other
when detected ! Among practitioners,
we have not heard a single dissentient
voice as to this effect which the writings
of the Lancet are calculated to produce.
Kut we shall come a little closer to the
point, and prove to a demonstration, the
inconsequences of the Lancet's reasoning.
This instrument; this " scarificator," in
its rage to excoriate an hospital surgeon,
has trod under foot and traduced three
General Practitioners?and then,
with unparalleled effrontery, tells these
last, that the whole procedure is deci-
dedly favourable to that class of the
profession to which they belong ! First
there was Mr. Jarman, who saw Mr.
Rolfe immediately after the accident?
who washed away the gravel?who found
a " hard moveable substance in the in-
terior, lying about an inch from the
knee-pan"?who attended the patient
home, helped Mr. Stanley to examine the
limb, and applied himself the splints.
Now we do not attach the slightest
blame to Mr. Jarman for not discovering
the true nature of the accident?but, in
the name of justice, is he not as much
responsible for the mistake as Mr. Stanley
?must not all censures levelled by the
Lancet against the one, fall equally on
the other, in the minds of the public?
Then, again, there was Mr. Janet, whom
we happen to know, and who is an experi-
enced and able general practitioner. He
attended with Mr. Stanley?but he could
not ascertain the precise nature of the
hard substance near the patella, and he,
too, is included in the sweeping accusa-
tions brought against the hospital sur-
geon. We now come to Mr. Lilly. This
gentleman candidly acknowledged in
court, that, after several examinations?
" he felt the hard substance, and thought
it was bone."?Lancet, No. 234, p. 746.
It is true that Mr. Lilly discovered his
mistake, when the flint perforated the
skin, and displayed its real character; but
surely there was nothing in this to ground
a contrast between his judgment and that
of Mr. Stanley. Thus, then, we see three
GENERAL PRACTITIO NERS, and OIIC HOS-
PITAL surgeon, all involved in one ge-
neral charge of ignorance or incapacity,
(for where all were concerned in the case,
and neither of them discovei'ed the stone
till it partly came through the skin, all
are necessarily included in the accusation,
whether ill or well founded) and yet this
instrument of j ustice insults the under-
standings of its readers, by asserting that,
in damning one out of the four, the other
three must be benefited !
An impartial examination of the evi-
dence, then, as well of the general prac-
titioners who actually saw the case, as
the distinguished surgeons who heard of
it, leads us to infer, that the obscurity of
the accident would have prevented the
generality of surgeons, of whatever deno-
mination, from ascertaining, with any de-
gree of certainty, the true nature of the
case, before the flint made its way through
the skin.* Two of the medical attendants
(Mr. Stanley and Mr. Janet) we know
personally and professionally; and, at the
risk of another action for libel, we declare
our firm conviction, that they are not in-
ferior to that celebrated, experienced, and
successful surgeon, Thomas WAKLEvhim-
self, by whose advice, in opposition to that
of Sir Astley Cooper, Mr. Lambert per-
formed his renowned operation, now mak-
ing the tour of Europe, and which enabled
the said Mr. Lambert (ultimately) to re-
move, without pain or haemorrhage, se-
veral inches of a troublesome and mis-
shapen carotid, as thousands of passen-
gers along the Strand can verify by oath.
We have now, we think, proved, to a de-
monstration, that the attempt to draw
public odium on Mr. Stanley, must in-
* Had we been called on for an opinion
upon oath, the above is that which we would
have given. A mistake was made by all
concerned in this case, as the event
shewed?and the probability is, that the
same mistake would have been made by
others?even by those who so loudly cry
o.ut shame! We neither censure nor ap-
plaud the evidence given by the hospital
surgeons on this trial?we only state what
we would ourselves have offered. It is
probable that a more moderate and diffi-
dent tone in the witnesses would have
been better for the defendant.
552 Periscope ; or, Circumspective Review. [Match 8
evitably draw public odium on all classes
of the profession, not even exempting that
class which has the misfortune of being
selected by the most scurrilous publi-
cation that ever issued from the press,
as the peculiar object of its mean syco-
phancy and nauseating adulation. That
class stands not in need of such an advo-
cate as the Lancet?whose censure is
applause?whose praise is poison !

				

